# The Magnetic Field Freezes the Mercedes–Benz Water Model

**DOI:** 10.3390/e25121618

**Published:** 2023-12-04

**Authors:** Tomaz Urbic

**Affiliations:** Faculty of Chemistry and Chemical Technology, University of Ljubljana, Večna Pot 113, SI-1000 Ljubljana, Slovenia; tomaz.urbic@fkkt.uni-lj.si

**Keywords:** water, magnetic field, anomalies

## Abstract

In this study, we investigate the impact of magnetic fields on the structural and thermodynamic properties of water. To accomplish this, we employed the Mercedes–Benz (MB) model, a two-dimensional representation of water using Lennard–Jones disks with angle-dependent interactions that closely mimic hydrogen bond formation. We extended the MB model by introducing two charges to enable interaction with the magnetic field. Employing molecular dynamics simulations, we thoroughly explored the thermodynamic properties concerning various magnetic flux intensities. As a result, we observed that under a weak magnetic flux, the property of water remained unaltered, while a stronger flux astonishingly led to the freezing of water molecules. Furthermore, our study revealed that once a specific flux magnitude was reached, the density anomaly disappeared, and an increase in flux caused the MB particles to form a glassy state.

## 1. Introduction

Water, the most crucial substance on Earth, holds unparalleled significance for life. Under ambient conditions, it exists in the liquid state and serves as a fundamental component in the realms of physics, chemistry, biology, and geoscience. It participates in various cycles and becomes an integral part of other compound cycles. What makes water truly remarkable are its unique properties, rendering it the most anomalous compound on our planet. Evidently, water exhibits numerous macroscopic anomalies, largely attributed to its exceptional ability to form up to four hydrogen bonds, a feature that surpasses the non-directional interactions typically observed in simple liquids. The prevalence of hydrogen bonds leads to an array of extraordinary behaviours, some of the most notable being increased density upon melting, reduced viscosity under pressure, density maxima at 4 °C, and high surface tension, among many others [1,2].

Electric and magnetic fields play significant roles in modifying the properties of water and other compounds. While the influence of electric fields has been extensively studied [3,4,5], the impact of magnetic fields remains relatively less explored. Electric fields may arise from ions and polar molecules dissolved in water, or they can be externally applied, such as through surface effects, like cracks in crystals or electrodes. In aqueous solutions, ions form hydration shells by reorienting neighbouring water molecules, and some ions can even hydrolyse water molecules.

On the other hand, the changes in water properties induced by a magnetic field represent an intriguing and important research area that still lacks comprehensive understanding, despite being studied for nearly a century [6,7,8]. Further exploration of the effects of magnetic fields on water properties holds promising potential for advancing our comprehension of these phenomena. Chang and Weng [9] conducted molecular dynamics simulations based on a flexible three-centered water model to investigate the effects of applying a magnetic field with strengths ranging from 1 to 10 T on the structure of liquid water. Their results demonstrated a slight increase in the number of hydrogen bonds formed in the water molecules as the strength of the magnetic field increased. Additionally, analysis of the water’s structure, using the radial distribution function of the water molecules, revealed enhanced stability and a greater propensity for forming hydrogen bonds when subjected to a magnetic field. Many experiments [6,7,8] show that water may be magnetized by a magnetic field, even though the magnetized effect is small. When water is exposed to a magnetic field, it undergoes changes in properties, including thermodynamics and mechanics. The dielectric constant, viscosity, surface tension, melting and boiling points, and electric conductivity are altered in a magnetic field compared to those of pure water outside of fields, but additional experimental studies must be undertaken in order to advance this field.

There are two ways to study water using different models. The first is to construct very detailed models having a wide range of applications but which can, in many cases, be computationally demanding [10,11,12,13]. For example, the TIP3P water forcefield was employed to simulate the structural and thermodynamic properties of an aqueous DNA solution [14]. The SPC/E water model can be used to explore the dynamics of peptides in bulk water [15]. The TIP4P water forcefield was used to study phase equilibria of aqueous systems [16]. To obtain insight into the physical background of many properties of water and aqueous solutions one can use simple models. In our opinion, the simplest model with water-like properties is the so-called Mercedes–Benz (MB) model [17], which was originally proposed by Ben-Naim in 1971 [18,19] and is also called the BNMB (Ben-Naim–-Mercedes–Benz) model. Here, the molecules are two dimensional (2D) and because of this it is also called a 2D water-like toy model. The MB water molecules are presented as disks. The interaction between molecules is through a Lennard–Jones (LJ) interaction and an orientation-dependent hydrogen bonding (HB) interaction. For the HB interaction, the disks have three radial arms arranged as in the MB logo. There are a couple of reasons for our interest in simplified models: (1) We can more easily study the influence of the parameters in potential functions and gain insights that are not obtainable from all-atom computer simulations. Simpler models are more flexible in providing insights and illuminating concepts. Simple models do not require large computer resources, especially in 2D where 100 particles is equivalent to 1000 3D particles; (2) Since for simple models we can obtain more detailed properties in phase space, they can be used as polygons to develop and study more analytical theoretical methods. The MB model is one of the simplest models of an orientation-dependent liquid. As such, it can be used as a test-bed for developing analytical theories that might ultimately be useful for more realistic models. Another great advantage of the MB model is that the underlying physical principles can be more readily explored and visualized in two dimensions. For the MB model, extensive Monte Carlo simulations were performed and showed that the MB model could qualitatively predict the density anomaly, the minimum in the isothermal compressibility as a function of temperature, the large heat capacity, as well as the experimental trends for the thermodynamic properties of the solvation of nonpolar solutes [17,20,21,22]. Another simple model is the rose water model which was recently proposed as a mimic of the MB water model [23]. Similar to the MB model, rose waters are modeled as Lennard–Jones disks with an added hydrogen bonding potential, but the potential is simpler and the computer simulations run faster.

In this paper, we report an investigation of the interaction between bulk MB water molecules and magnetic fields of varying fluxes. This study is a continuation of our previous Monte Carlo simulation, in which we explored the interaction of the MB model with an electric field [24]. For this research, we utilized a modified version of the MB model previously introduced by Hribar et al. [24,25], making slight adjustments to it. We introduced a negative charge on one arm and a positive charge on the opposite side of the arm while maintaining the same distance between the charges as outlined in Hribar et al.’s modifications [25]. This configuration resulted in the center of mass and the center of the electric charges coinciding, simplifying the equations of motion. The original configuration of charges presented by Hribar et al. used different rotation axes for the magnetic field and the intermolecular potential.

## 2. The Model

**Figure 1:**
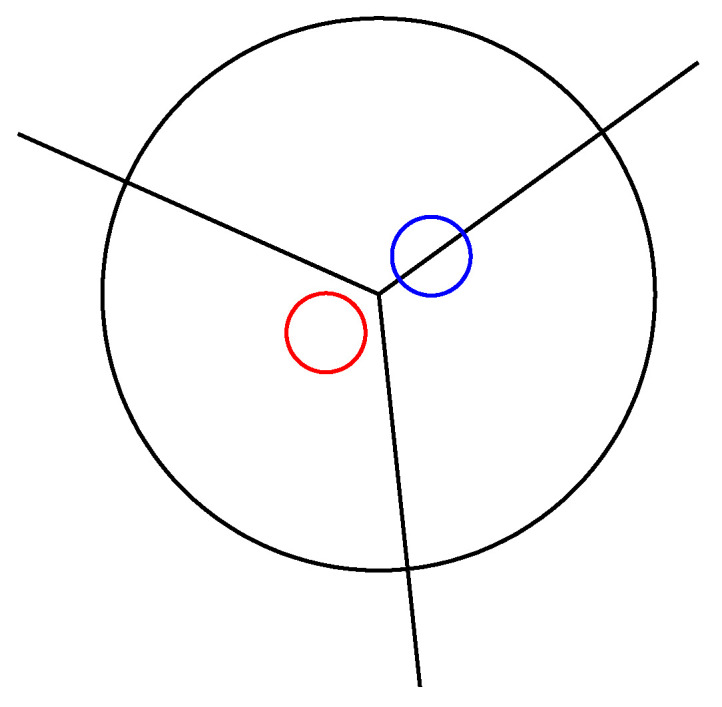
The MB particle with charges. The red circle shows a positive charge while the blue circle shows a negative charge for the interaction with the magnetic field.

The MB model was proposed by Ben-Naim in 1971 [18,19]. Within the model, the molecule is described as a 2D LJ disk with three attached arms which mimic the formation of the hydrogen bonds (See Figure 1). The angle between the pair arms is 120${}^{\circ }$ [17,18,19]. Two molecules interact by means of the Lennard–Jones (LJ) interaction and hydrogen-bonding (HB) angular-dependent interaction, which depends on the distance between the molecules and their orientation. The total interaction is calculated as

(1) $$U\left(\vec{X_{i}},\vec{X_{j}}\right)=U_{LJ}\left(r_{ij}\right)+U_{HB}\left(\vec{X_{i}},\vec{X_{j}}\right)$$

where $\vec{X_{i}}$ and $\vec{X_{j}}$ are the vectors of the orientation and position of the *i*-th and *j*-th molecules. The distance between the *i*-th and the *j*-th molecules is $r_{ij}$. The LJ term is enumerated in a standard way

(2) $$U_{LJ}\left(r_{ij}\right)=4\varepsilon_{LJ}\left[{\left(\frac{\sigma_{LJ}}{r_{ij}}\right)}^{12}-{\left(\frac{\sigma_{LJ}}{r_{ij}}\right)}^{6}\right],$$

by $\varepsilon_{LJ}$ and $\sigma_{LJ}$, being the contact distance and depth of the LJ interaction. The HB interaction is an aggregate of all interactions $U_{HB}^{kl}$ between the arms *k* and *l* of the molecules *i* and *j*, respectively.

(3) $$U_{HB}\left(\vec{X_{i}},\vec{X_{j}}\right)=\sum_{k,l=1}^{3}U_{HB}^{kl}\left(r_{ij},\theta_{i},\theta_{j}\right).$$

where $\theta_{i}$ and $\theta_{j}$ are the angles of orientation of the *i*-th and *j*-th molecules. The HB contribution is the product of Gaussian functions, which depends on the orientation of each molecule and the distance

(4) $$U_{HB}^{kl}\left(r_{ij},\theta_{i},\theta_{j}\right)=\varepsilon_{HB}G\left(r_{ij}-r_{HB}\right)G\left(\vec{i_{k}}\vec{u_{ij}}-1\right)G\left(\vec{j_{l}}\vec{u_{ij}}+1\right)$$

(5) $$U_{HB}^{kl}\left(r_{ij},\theta_{i},\theta_{j}\right)=\varepsilon_{HB}G\left(r_{ij}-r_{HB}\right)G\left(\cos\left(\theta_{i}+\frac{2\pi }{3}\left(k-1\right)\right)-1\right)G\left(\cos\left(\theta_{j}+\frac{2\pi }{3}\left(l-1\right)\right)+1\right).$$

G(x) is an un-normalized Gaussian function:

(6) $$G\left(x\right)=\exp\left(-\frac{x^{2}}{2\sigma^{2}}\right).$$

$\varepsilon_{HB}$ is the maximum energy of the hydrogen bond, while $r_{HB}$ is the distance at which the hydrogen bond is formed. $\vec{u_{ij}}$ is the unit vector in the direction of $\vec{r_{ij}}$. $\vec{i_{k}}$ and $\vec{j_{l}}$ are the unit vectors of the *k*-th arm of the *i*-th molecule and the *l*-th arm of the *j*-th molecule. When two molecules are at a distance $r_{HB}$ and their interacting arms are parallel and pointing towards each other’s centres, the interaction between the molecules is the strongest. The same units as in previous studies were used: the energies were expressed in $|\varepsilon_{HB}|$ and the lengths in $r_{HB}$. Thus, the energy parameter for the hydrogen-bond, $\varepsilon_{HB}$, was −1, and the hydrogen-bond length equalled 1. The same width parameter σ=0.085 was used for both the distance and the angle deviation of a hydrogen bond. The parameters of the LJ potential were set to: $\varepsilon_{LJ}=0.1\left|\varepsilon_{HB}\right|$ and $\sigma_{LJ}=0.7r_{HB}$. For the interaction of the molecules with the magnetic field, we changed the modified MB model of Hribar et al. [25], which includes an electrostatic dipole for interaction with external fields. A single negative charge ($e_{-}=-e_{0}$) is put onto one of the H-bonding arms, at a distance $0.0825r_{HB}$ from the center (See Figure 1). A single positive charge $e_{+}=e_{0}$ is put on the opposite site of the arm at the same distance from the center. The distance between the charges is $r_{c}=0.165r_{HB}$, as in the initial paper [24,25]. The other arms are uncharged. This dipole is added only for the interaction between the water molecules and the magnetic field; the water–water interaction is the same as previously described. This kind of approximation was shown to work effectively for water in an electric field [24,25]. The size of the dipole moment is $0.165e_{0}r_{HB}$. To describe the rotation, we put 0.7 of the total mass of the molecule in the center and on each arm at a distance $0.35r_{HB}$ 0.1 of the total mass.

## 3. Molecular Dynamics

We performed molecular dynamics (MD) simulations to determine the properties of the MB model in the magnetic field of different fluxes using the code developed by our group. The MD simulations were carried out in an NpT ensemble. To mimic the macroscopic system, we used the minimum image convention and periodic boundary conditions. The equations of motion were integrated using a simple velocity Verlet algorithm with a time step between $10^{-6}$ and $10^{-3}$ ($t^{*}=t\sqrt{\frac{\epsilon_{HB}}{mr_{HB}^{2}}}$). At the beginning, the system was equilibrated by simulation of a minimum of 100,000 steps in length, then the sampling phase of the simulation was performed in a minimum of 20 series, where each series was a minimum of 100,000 steps long. To maintain constant temperatures and pressures, we employed a Berendsen thermostat [26], with the time constant equal to 0.1, and a Berendsen barostat with the same time constant as for the thermostat. The thermostat and the barostat performed well in all the studied phase points. In the simulation box, we had 100–400 MB molecules at all times. We checked that there were no size effects. The initial positions of the molecules were randomly chosen so that there was no overlap between the molecules. The initial velocities were drawn from the Maxwell–Boltzmann distribution. During the sampling phase, the thermodynamic quantities (heat capacity at constant pressure $c_{p}$, thermal expansion coefficient α, and the isothermal compressibility $\kappa_{T}$) were calculated as statistical averages of the enthalpy (*H*), the volume (*V*) and the fluctuations [27,28]

(7) $$\begin{array}{c} c_{p}=\frac{<H^{2}>-<H{>}^{2}}{Nk_{B}T^{2}}\\ \kappa_{T}=\;\frac{<V^{2}>-<V{>}^{2}}{k_{B}T<V>}\\ \alpha =\;\frac{<VH>-<V><H>}{k_{B}T^{2}<V>} \end{array}$$

where *N* is the number of particles in the system and *T* is the temperature. The interaction with a magnetic field perpendicular to the plane in which the MB molecules could move was treated in the following way: When an electric dipole moves within the magnetic field, it induces both a force ($F_{mx},F_{my}$) and a torque ($M_{m}$) acting on the MB particle, which are calculated as

(8) $$F_{mx}=eB\omega r_{c}cos\phi ,$$

(9) $$F_{my}=eB\omega r_{c}sin\phi ,$$

(10) $$M_{m}=-eBr_{c}\left(v_{x}cos\phi +v_{y}sin\phi \right),$$

where $e=|e[-]|=e_{+}$ is the absolute value of the charge, *B* is the magnetic flux density, and ϕ is the orientation of the MB particle. $v_{x}$ and $v_{y}$ are the components of the velocities of the center of mass of the MB particle.

## 4. Results and Discussion

All the findings are presented using reduced units for enhanced clarity and comparability. To achieve this, we utilized the HB energy parameter $\epsilon_{HB}$ as the normalization factor for both the temperature and the excess internal enthalpy, yielding reduced variables ($A^{*}=\frac{A}{|\varepsilon_{HB}|}$ and $T^{*}=\frac{k_{B}T}{|\varepsilon_{HB}|}$, respectively). Additionally, the distances were normalized based on the characteristic length of the hydrogen bond, $r_{HB}$, denoted as $r^{*}=\frac{r}{r_{HB}}$ and the time denoted as $t^{*}=t\sqrt{\frac{|\varepsilon_{HB}|}{mr_{HB}^{2}}}$. Furthermore, the flux of the magnetic field was normalized as $B^{*}=\frac{e_{0}Br_{HB}}{\sqrt{m|\varepsilon_{HB}|}}$, ensuring that the results were independent of the specific units. For instance, a value of $B^{*}=100$ translates to approximately $B=7\times 10^{7}$ Vs/m${}^{2}$, depending on the experimental values of $r_{HB}$ and $\varepsilon_{HB}$.

**Figure 2:**
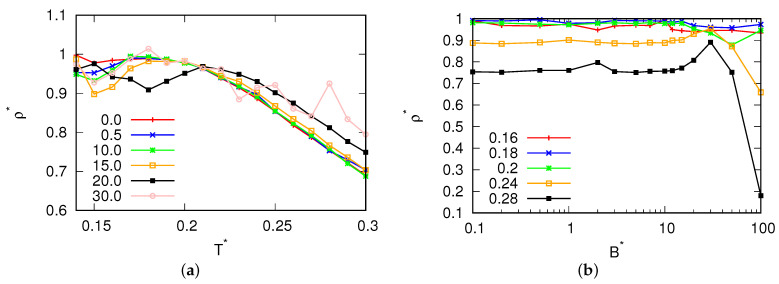
Density as a function of (**a**) the temperature for different magnetic fluxes at pressure $p^{*}=0.19$ and (**b**) the magnetic field flux.

**Figure 3:**
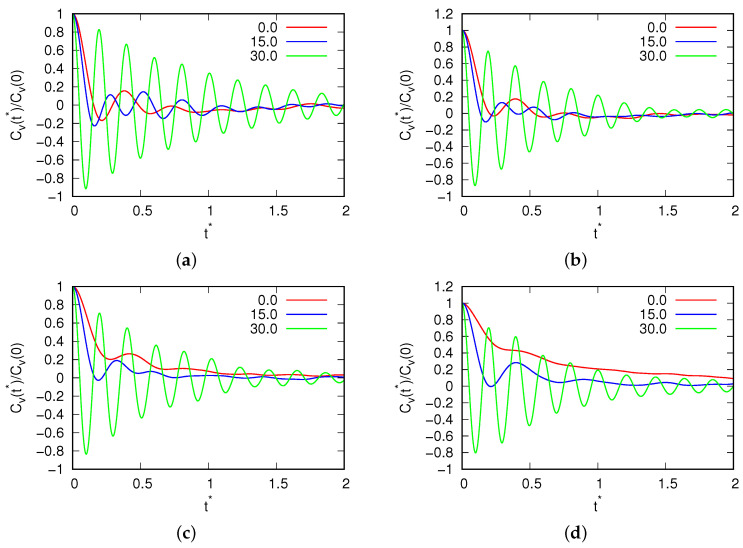
Velocity autocorrelation function of water molecules for (**a**) $T^{*}=0.15$; (**b**) $T^{*}=0.18$; (**c**) $T^{*}=0.24$; and (**d**) $T^{*}=0.32$ for different fluxes of the magnetic field.

**Figure 4:**
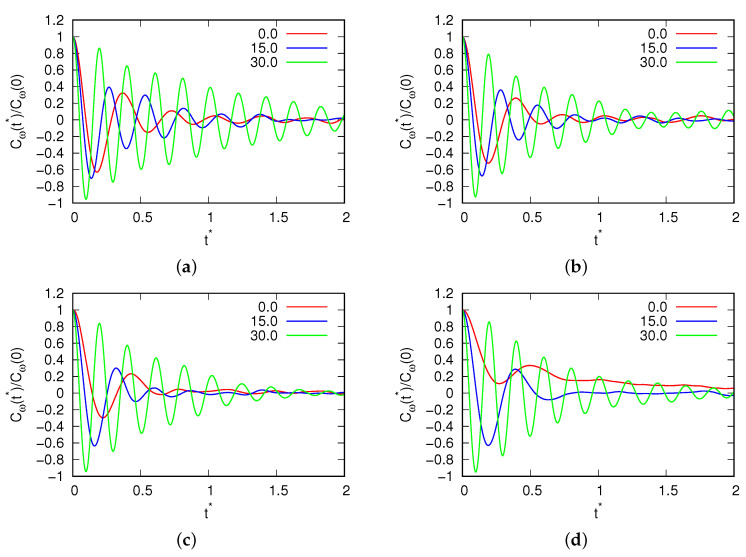
Angular velocity autocorrelation function of water molecules for (**a**) $T^{*}=0.15$; (**b**) $T^{*}=0.18$; (**c**) $T^{*}=0.24$; and (**d**) $T^{*}=0.32$ for different fluxes of the magnetic field.

The study began by investigating the temperature dependence of the density at a pressure of $p^{*}=0.19$, with varying magnetic field fluxes. The resulting density data are depicted in Figure 2. The error of the results in the liquid range is the size of the points, while when crystallization occurs, the results depend on the amorphous phase formed by the system. This pressure was deliberately selected to maintain consistency with previous MB simulations (as reported in [17]), where the MB model demonstrated a density anomaly. At this pressure, the MB molecules undergo freezing at approximately $T^{*}=0.15$, forming a low-density hexagonal crystal phase. At magnetic field fluxes below $B^{*}<10.0$, there was no discernible impact on either the density or the position of the density anomaly. This suggests that within this range, the system exhibited stability in terms of anomaly location. However, as we transitioned to intermediate fluxes, specifically those ranging from $10.0<B^{*}<20.0$, noteworthy changes were observed in our simulation results. We observed a displacement in the density anomaly towards higher temperatures with an increase in density. This phenomenon can be attributed to the influence of a more robust hydrogen bond network. Additionally, we noted an increase in the melting point under these conditions, which is seen where crystallization takes place in the same figure. This is seen where the curve breaks at lower temperatures. Subsequently, the water molecules froze into a glassy state. This freezing phenomenon is evident in the velocity and angular velocity autocorrelation functions, as presented in Figure 3 and Figure 4, respectively. These figures reveal the presence of solid-phase structures (long-range osculations which are characteristic of solid phases) at both low and high temperatures for the high magnetic field. However, such long-range structures were absent for small magnetic field fluxes where the system was in a liquid state.

**Figure 5:**
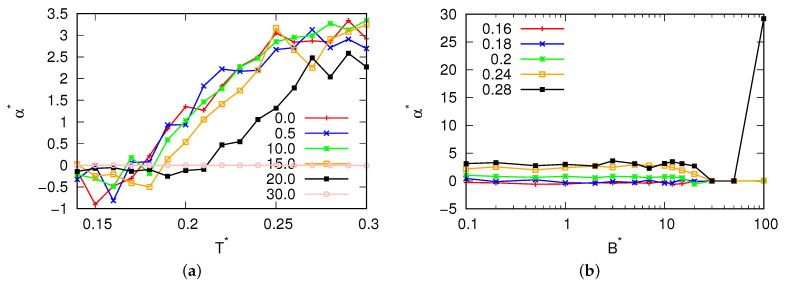
Thermal expansion coefficient as a function of (**a**) the temperature for different magnetic fluxes at pressure $p^{*}=0.19$ and (**b**) the magnetic field flux.

**Figure 6:**
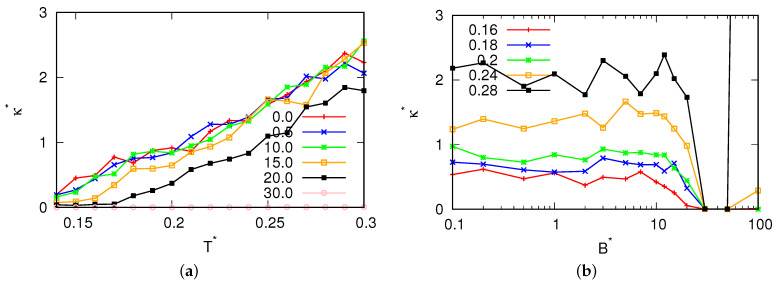
Isothermal compressibility as a function of (**a**) the temperature for different magnetic fluxes at pressure $p^{*}=0.19$ and (**b**) the magnetic field flux.

**Figure 7:**
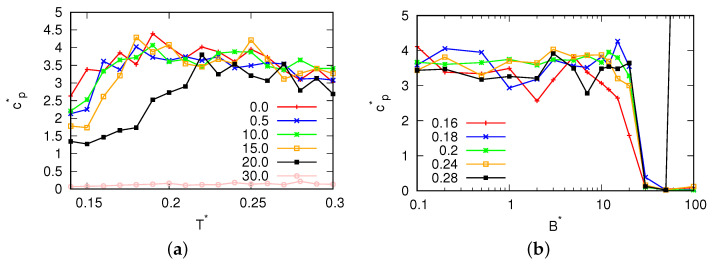
Heat capacity at constant pressure as a function of (**a**) the temperature for different magnetic fluxes at pressure $p^{*}=0.19$ and (**b**) the magnetic field flux.

**Figure 8:**
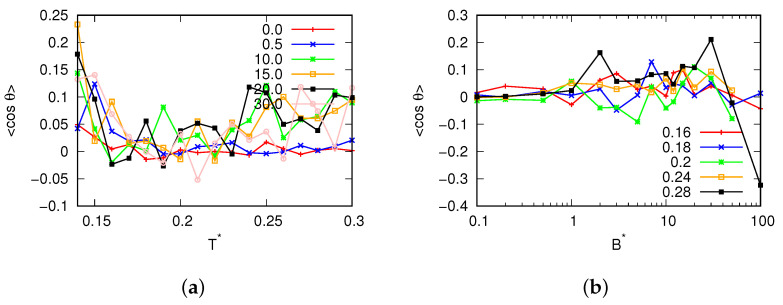
The averaged cosine of the angle of water molecules with respect to the x-axis as a function of (**a**) the temperature for different magnetic fluxes at pressure $p^{*}=0.19$ and (**b**) the magnetic field flux.

**Figure 9:**
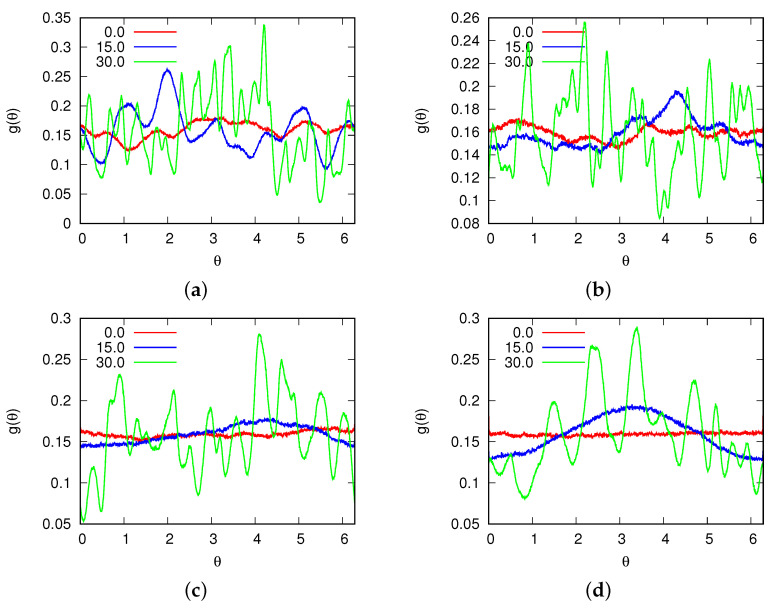
Distribution of orientations of water molecules for (**a**) $T^{*}=0.15$; (**b**) $T^{*}=0.18$; (**c**) $T^{*}=0.24$; and (**d**) $T^{*}=0.32$ for different fluxes of the magnetic field.

For magnetic field fluxes smaller than $B^{*}<25.0$, the presence of a density anomaly was observed. Significantly, to the left of the density maxima (refer to Figure 5), we observed negative values for the thermal expansion coefficient. Additionally, with increase in the magnetic flux, we observed a change in the location where the thermal expansion coefficient reached zero, confirming the migration of the density maxima towards higher temperatures. Additionally, for fluxes where the MB model was in a glassy state (Figure 6), the compressibility of the MB model was found to be almost 0. These fluxes also exhibited low heat capacity (Figure 7). In Figure 8, the cosine of the orientation angle of the MB particles is plotted, which is indicative of the polarization and is proportional to the averaged cosine of the model’s angle due to dipole alignment. Interestingly, we observed that this quantity oscillated around 0 for all fluxes. This suggests that the magnetic field does not preferentially orient the dipoles of water with respect to the x-axis. Nevertheless, the alignment of the dipoles is further confirmed by the angular distribution of the water molecules, as shown in Figure 9, although we did observe small preferred orientations for mid-range fluxes.

**Figure 10:**
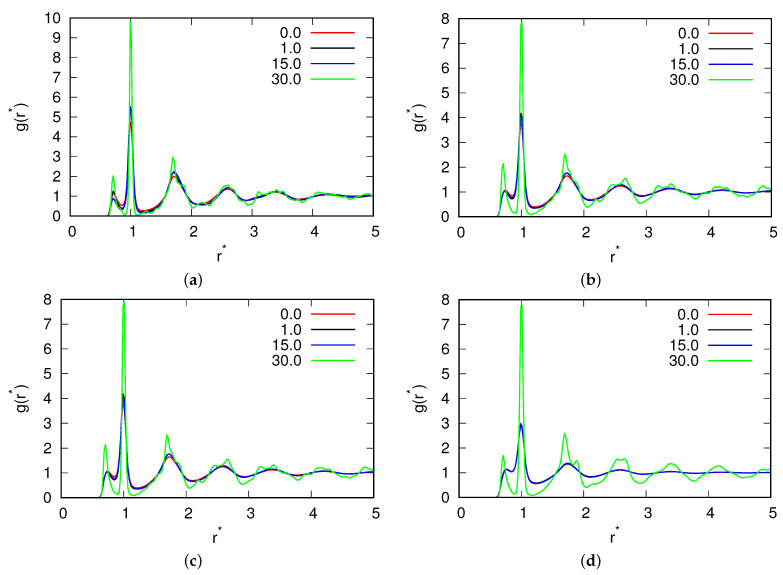
Pair correlation function between water molecules for (**a**) $T^{*}=0.15$; (**b**) $T^{*}=0.18$; (**c**) $T^{*}=0.24$; and (**d**) $T^{*}=0.32$ for different fluxes of the magnetic field.

**Figure 11:**
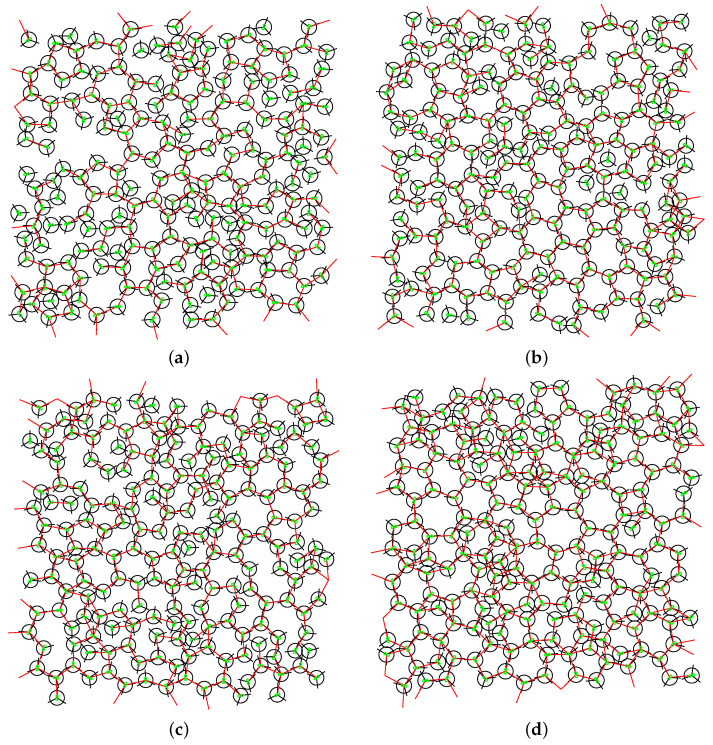
Snapshots of the system for different fluxes of the magnetic field field at pressure $p^{*}=0.19$ and temperature $T^{*}=0.18$. Red lines connect MB molecules that form HBs. Green lines are plotted charges for interaction with the magnetic field. Magnetic fluxes are (**a**) $B^{*}=0.0$; (**b**) $B^{*}=1.0$; (**c**) $B^{*}=15.0$ and (**d**) $B^{*}=30.0$.

**Figure 12:**
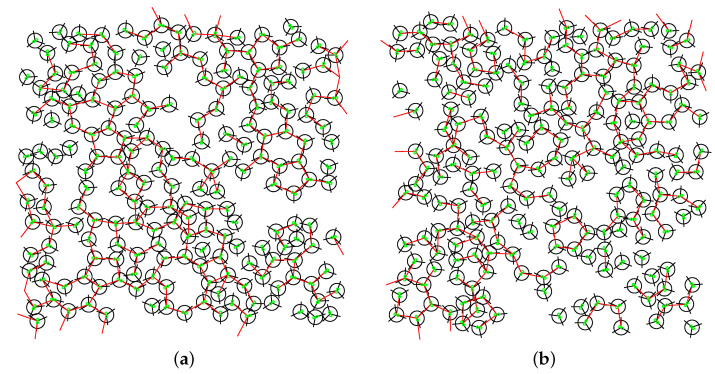

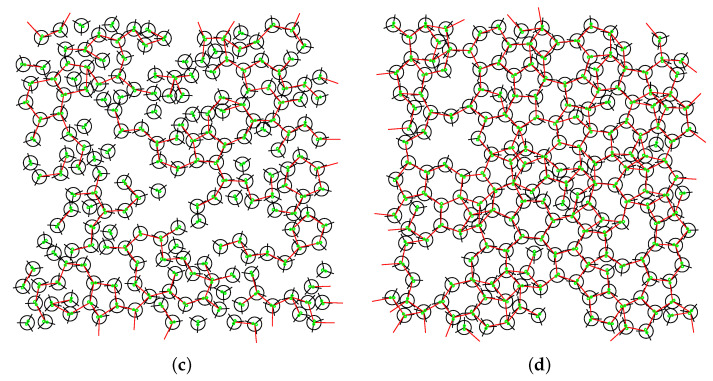
Snapshots of the system for different fluxes of the magnetic field field at pressure $p^{*}=0.19$ and temperature $T^{*}=0.24$. Red lines connect MB molecules that form HBs. Green lines are plotted charges for interaction with the magnetic field. Magnetic fluxes are (**a**) $B^{*}=0.0$; (**b**) $B^{*}=1.0$; (**c**) $B^{*}=15.0$ and (**d**) $B^{*}=30.0$.

Towards the end of our study, we conducted a thorough examination of the pair correlation functions (Figure 10) and visualized snapshots of the molecular configurations (Figure 11 and Figure 12). The pair correlation functions provided compelling evidence of the molecular freezing phenomenon occurring at high magnetic fluxes, corroborating our earlier observations. Furthermore, for mid-range fluxes, we observed a strengthening of the hydrogen bond network, which was reflected in the higher peaks evident in the pair correlation functions due to increased hydrogen bonding. The snapshots of the molecular configurations provided a direct visualization of the system’s behaviour under different magnetic flux conditions. At high fluxes, the snapshots clearly illustrated the frozen state of the water molecules, validating the freezing phenomenon observed in other analyses. For mid-range fluxes, the snapshots vividly displayed the formation and enhancement of hydrogen bonds, supporting the findings from the pair correlation functions. Both the pair correlation functions and the snapshots of the molecular configurations offered complementary insights into the effects of the magnetic fluxes on the system. They provided visual evidence of freezing at high fluxes and the reinforcement of the hydrogen bond network at mid-range fluxes, emphasizing the profound influence of magnetic fields on the structural and dynamical properties of the system.

## 5. Conclusions

In this investigation, the impact of magnetic fields on the structural and thermodynamic properties of MB water was explored using molecular dynamics simulations. The Mercedes–Benz (MB) model was used. The MB model is a two-dimensional representation where water particles embody Lennard–Jones disks, featuring angle-dependent interactions that ingeniously mimic the intricate formation of hydrogen bonds and exhibit water-like anomalies. We enhanced the original MB model by introducing two charges, thereby facilitating interaction with the magnetic field. Our results revealed interesting dynamics concerning various magnetic flux intensities. For instance, under the influence of a very small magnetic flux ($B^{*}<0.5$), no discernible changes in the position of the phase transitions or the water’s thermodynamics were observed, suggesting that these modest fluxes do not substantially alter the fundamental nature of water. Intriguingly, as we increased the magnetic intensity to strong flux levels ($B^{*}=20.0$), a transformation occurred, as the water molecules swiftly transitioned into a glassy structure. The implications of this revelation are profound, as it unveils the magnetic field’s remarkable ability to impose substantial changes on water’s structural properties. Furthermore, we explored the middle range of magnetic fluxes, which remarkably augmented the water’s hydrogen bond network. These findings provide vital insights into the intricate relationship between magnetic fields and water’s behaviour at the molecular level.

## Data Availability

Data are contained within the article.

## References

[1] Brini E., Fennell C.J., Fernandez-Serra M., Hribar-Lee B., Luksic M., Dill K.A. (2017). How Water’s Properties Are Encoded in Its Molecular Structure and Energies. Chem. Rev..

[2] Gallo P., Amann-Winkel K., Angell C.A., Anisimov M.A., Caupin F., Chakravarty C., Lascaris E., Loerting T., Panagiotopoulos A.Z., Russo J. (2016). Water: A Tale of Two Liquids. Chem. Rev..

[3] Bartlett J.T., Heuval A.P.v., Mason B.J. (1963). The growth of ice crystals in an electric field. Z. Angew. Math. Phys..

[4] Choi E.-M., Yoon Y.-H., Lee S., Kang H. (2005). Freezing Transition of Interfacial Water at Room Temperature under Electric Fields. Phys. Rev. Lett..

[5] Aragones J.L., MacDowell L.G., Siepmann J.I., Vega C. (2011). Phase Diagram of Water under an Applied Electric Field. Phys. Rev. Lett..

[6] Wang Y., Wei H., Li Z. (2018). Effect of magnetic field on the physical properties of water. Results Phys..

[7] Pang X., Deng B. (2008). Investigation of changes in properties of water under the action of a magnetic field. Sci. China Ser. G-Phys. Mech. Astron..

[8] Chibowski E., Szczes A. (2018). Magnetic water treatmente—A review of the latest approaches. Chemosphere.

[9] Chang K.-T., Weng C.-I. (2006). The effect of an external magnetic field on the structure of liquid water using molecular dynamics simulation. J. Appl. Phys..

[10] Jorgensen W.L., Chandrasekhar J., Madura J.D., Impey R.W., Klein M.L. (1983). Comparison of simple potential functions for simulating liquid water. J. Chem. Phys..

[11] Nezbeda I. (1997). Simple short-ranged models of water and their application. A review. J. Mol. Liq..

[12] Guillot B. (2002). A reappraisal of what we have learnt during three decades of computer simulations on water. J. Mol. Liq..

[13] Vega C., Abascal J.L.F., Conde M.M., Aragones J.L. (2009). What ice can teach us about water interactions: A critical comparison of the performance of different water models. Faraday Discuss.

[14] Fujimoto S., Yu Y.-X. (2010). Effect of electrolyte concentration on DNA A-B conformational transition. An unrestrained molecular dynamics simulation study. Chin. Phys. B.

[15] Fonseca B., Freeman C.L., Collins M.J. (2022). Conformational analysis and water dynamics: A molecular dynamics study on the survival of a beta-lactoglobulin peptide in the archaeological record. Chem. Phys..

[16] Vishnyakov A., Weathers T., Hosangadi A., Chiew Y.C. (2020). Molecular models for phase equilibria of alkanes with air components and combustion products I. Alkane mixtures with nitrogen, CO_2_ and water. Fluid Phase Equilibria.

[17] Silverstein K.A.T., Haymet A.D.J., Dill K.A. (1998). A Simple Model of Water and the Hydrophobic Effect. J. Am. Chem. Soc..

[18] Ben-Naim A. (1971). Statistical Mechanics of “Waterlike” Particles in Two Dimensions. I. Physical Model and Application of the Percus–Yevick Equation. J. Chem. Phys..

[19] Ben-Naim A. (1972). Statistical mechanics of water-like particles in two-dimensions. Mol. Phys..

[20] Southall N.T., Dill K.A. (2000). The Mechanism of Hydrophobic Solvation Depends on Solute Radius. J. Phys. Chem. B.

[21] Silverstein K.A.T., Haymet A.D.J., Dill K.A. (2001). Hydrophobicity in a simple model of water: Entropy penalty as a sum of competing terms via full, angular expansion. J. Chem. Phys..

[22] Dias C.L., Hynninen T., Ala-Nissila T., Foster A.S., Karttunen M. (2011). Hydrophobicity within the three-dimensional Mercedes-Benz model: Potential of mean force. J. Chem. Phys..

[23] Williamson C.H., Hall J.R., Fennell C.J. (2017). Two-dimensional molecular simulations using rose potentials. J. Mol. Liq..

[24] Urbic T. (2023). The electric field changes the anomalous properties of the Mercedes Benz water model. Phys. Chem. Chem. Phys..

[25] Hribar B., Southall N.T., Vlachy V., Dill K.A. (2002). How Ions Affect the Structure of Water. J. Am. Chem. Soc..

[26] Berendsen H.J.C., Postma J.P.M., van Gunsteren W.F., Di Nola A., Haak J.R. (1984). Molecular dynamics with coupling to an external bath. J. Chem. Phys..

[27] Hansen J.P., McDonald I.R. (1986). Theory of Simple Liquids.

[28] Frenkel D., Smit B. (2000). Molecular Simulation: From Algorithms to Applications.

